# Forearm Injury Associated with Compound Presentation and Prolonged Labour

**Published:** 2015-07-01

**Authors:** Chun-Sui Kwok, Claire-Lise Judkins, Matthew Sherratt

**Affiliations:** 1 Oxford University Hospitals NHS Trust, UK; 2 Hopitaly Vaovao Mahafaly, Mandritsara, Madagascar

A 25-year-old pregnant female presented at term to her local district general hospital in Madagascar after 28 hours of labour, having travelled 30km on foot. On initial assessment, compound presentation involving the hand was discovered, with prolapse of an extended forearm of the foetus. The forearm was swollen with signs of venous congestion. Vaginal delivery was clearly not appropriate and emergency Caesarean section was performed within the hour. Upon delivery, the infant was noted to have gross swelling, ecchymosis and cherry red discolouration in the right forearm. Mother and child remained otherwise well. The injury was treated conservatively without dressings, leaving the arm to open air. Routine prophylactic antibiotics were administered to the infant given the history of prolonged rupture of membranes. On post-delivery day 3, two tense serous bullae had formed on the flexor aspect of the forearm (Fig. 1). The forearm made almost complete recovery by post-delivery day 10.

**Figure:**
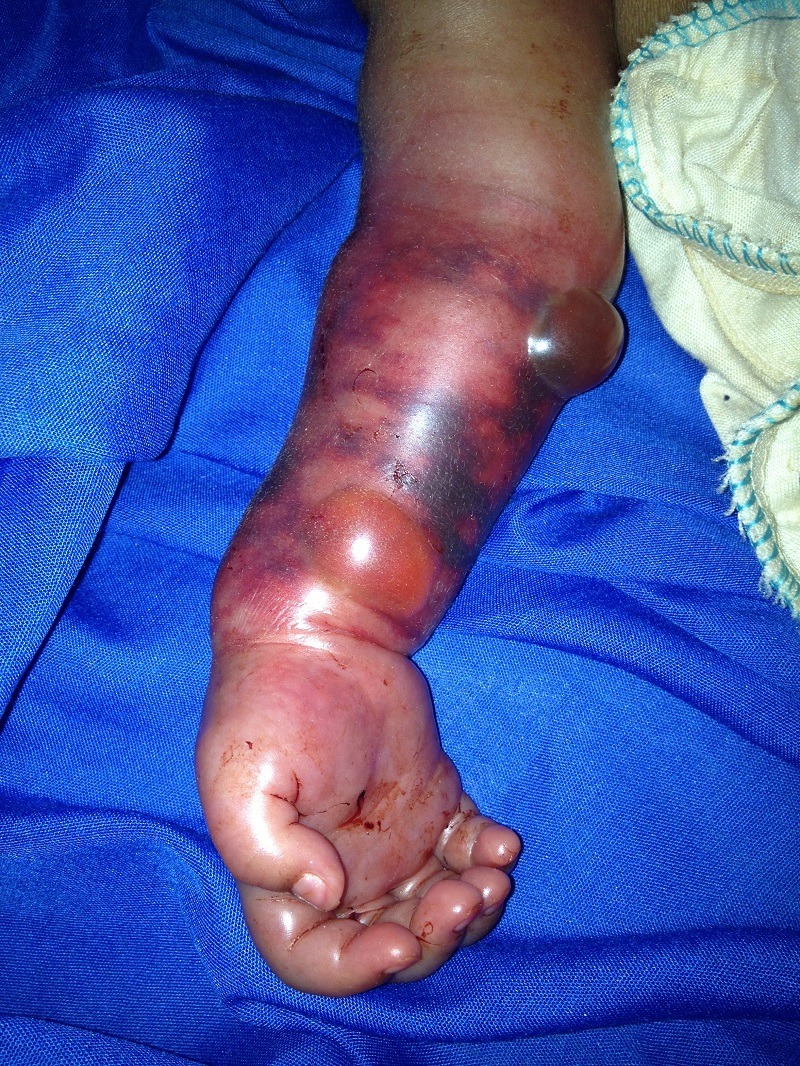
Figure 1 : Severe ecchymosis, oedema and bullae formation, day 3 post-delivery.

Compound presentations involving hand or foot complicate 1 in 300 to 1 in 1300 deliveries.[1, 2] Foetal-pelvic disproportion, polyhydramnios, and premature rupture of membranes may predispose to compound presentation. In normally progressing labour, the situation may be observed initially as the foetus may retract the extremity as labour progresses allowing for successful vaginal delivery. In protracted labour, compound presentation may progress to prolapse of the extremity which may lead to soft tissue injury to the limb if no intervention is made. There has been one other reported case of forearm injury following compound presentation, which unfortunately progressed to ischaemic necrosis requiring amputation.[3]

## Footnotes

**Source of Support:** Nil

**Conflict of Interest:** Nil

